# Distinctive Roles for α7*- and α9*-Nicotinic Acetylcholine Receptors in Inflammatory and Autoimmune Responses in the Murine Experimental Autoimmune Encephalomyelitis Model of Multiple Sclerosis

**DOI:** 10.3389/fncel.2017.00287

**Published:** 2017-09-22

**Authors:** Qiang Liu, Paul Whiteaker, Barbara J. Morley, Fu-Dong Shi, Ronald J. Lukas

**Affiliations:** ^1^Division of Neurobiology, Barrow Neurological Institute Phoenix, AZ, United States; ^2^Boys Town National Research Hospital Omaha, NE, United States

**Keywords:** auto-immunity, cholinergic anti-inflammatory pathway, experimental autoimmune encephalomyelitis, inflammation, multiple sclerosis, nicotinic acetylcholine receptors

## Abstract

Previous studies have demonstrated immunosuppressive and anti-inflammatory effects of nicotine, including in the experimental autoimmune encephalomyelitis (EAE) model in mice of some forms of multiple sclerosis (MS). Other studies using knock-out (KO) mice have implicated nicotinic acetylcholine (ACh) receptors containing α7, α9, or β2 subunits (α7*-, α9*- or β2*-nAChR) in different, disease-exacerbating or disease-ameliorating processes. These outcomes are in harmony with gene expression analyses showing nAChR subunit mRNA in many classes of immune system cell types. Consistent with influences on disease status, predictable effects of nAChR subunit (and subtype) KO, or of nicotine exposure, are seen on immune cell numbers and distribution and on cytokine levels or other markers of immunity, inflammation, demyelination, and axonal degradation. Providing support for our hypotheses about distinctive roles for nAChR subtypes in EAE, here we have used direct and adoptive EAE induction and a nAChR subunit gene double knock-out (DKO) strategy. Immune cell expression of nAChR α9 subunits as protein is demonstrated by immunostaining of isolated CD4^+^, CD8^+^, CD11b^+^ and CD11c^+^ cells from wild-type (WT) mice, but not in cells from nAChR α9 subunit KO animals. Nicotine exposure is protective against directly-induced EAE in WT or α7/α9 DKO animals relative to effects seen in WT/vehicle-treated mice, but, remarkably, EAE is exacerbated in vehicle-treated α7/α9 DKO mice. Brain lesion volume and intra-cranial inflammatory activity similarly are higher in DKO/vehicle than in WT/vehicle-treated animals, although nicotine’s protective effects are seen in each instance. By contrast, in adoptive transfer studies, disease severity is attenuated and disease onset is delayed in recipients of splenocytes from WT animals treated with nicotine rather than with vehicle. Moreover, protection as seen in nicotine-treated WT animals is the same in recipients of splenocytes from nAChR α7/α9 DKO mice irrespective of their exposure to nicotine or vehicle. When combined with previous observations, these findings are consistent with disease exacerbation (or even induction) being mediated at least in part via α9*-nAChR in peripheral immune cells. They also suggest protective roles of central nervous system (CNS) α7*-nAChR. The results suggest that both α7*- and α9*-nAChR are potential targets of therapeutic ligands to modulate inflammation and autoimmunity.

## Introduction

It is widely recognized and undisputed that controlled inflammatory and immune responses play critical roles in maintenance of normal health by protecting against insults, in part by clearing damaged cells, compromised tissue areas and/or foreign substances (Weiner and Selkoe, 2002; Nikoopour et al., 2008; Viganò et al., 2012). Conversely, aberrant or excessive inflammatory or immune responses and anti-self, autoimmune activities contribute to or literally cause a variety of diseases, such as inflammatory bowel disease, arthritis and multiple sclerosis (MS; Wekerle, 1998; Davidson and Diamond, 2001; Weiner and Selkoe, 2002; Nikoopour et al., 2008; Franklin et al., 2012; Pierson et al., 2012; Viganò et al., 2012; Lassmann, 2013; Laveti et al., 2013). Our work reported here sought to illuminate new bases for possible improvements in treatment of MS and other diseases provoked by inflammation and hyper-immunity.

We and others have shown protection against inflammation and immune hyper-responsiveness in the murine experimental autoimmune encephalomyelitis (EAE) model of some forms of MS upon sustained exposure to nicotine (Nizri et al., 2009; Shi et al., 2009). These findings are consistent with other, prior work, indicating that nicotine exposure inhibits, for example, T cell differentiation (Sato et al., 1999; Matsunaga et al., 2001; Kuo et al., 2002; Middlebrook et al., 2002; Kawashima et al., 2007; Fujii et al., 2008), with long-recognized indications that nicotine has anti-inflammatory and immunosuppressive effects (Ulloa, 2005; Cloëz-Tayarani and Changeux, 2007; Filippini et al., 2012), and with the concept of a cholinergic anti-inflammatory system (Tracey, 2009), which is postulated to suppress inflammatory and immune responses by integrating signaling in the immune and nervous systems.

Our subsequent studies have confirmed those protective effects in the EAE model and have illuminated roles for different nicotinic acetylcholine receptor (nAChR) subtypes in specific features of the disease process or recovery and in protective effects of nicotine exposure (Piao et al., 2009; Shi et al., 2009; Hao et al., 2011, 2013; Simard et al., 2013). nAChR exist as pentamers of nAChR subunits that are encoded from a mammalian family of 16 different genes (Jensen et al., 2005; Lukas and Bencherif, 2006; Taly et al., 2009). nAChR thus also exist as a group of subtypes, each defined by their distinctive subunit composition. Subtypes have unique distributions across body organs, organ (brain) regions, cell types and even across sub-cellular domains (nerve cell soma, dendrites or axon terminals; pre-, post- or peri-synaptic), and each nAChR subtype also has unique features and characteristic responsiveness to acetylcholine (ACh) and nicotine. nAChR nomenclature is based on subunit composition to the extent that it is known or inferred, and an “*” is used to indicate a set of nAChR containing the specified subunits but that additional and unspecified subunits may be or are known to be partners in the receptor assembly (Lukas et al., 1999).

Prior studies pointed to important roles for α7*-nAChR in the cholinergic anti-inflammatory pathway (Wang et al., 2003; Shytle et al., 2004). Studies by us and others at first seemed consistent with a dominant role for α7*-nAChR in protective effects of nicotine in EAE (Nizri et al., 2009; Hao et al., 2011). In part, this is because the integrated disease response, assessed based on signs of limb and tail weakness in the EAE model, was attenuated by nicotine treatment in wild-type (WT) animals, but not in nicotine-treated nAChR α7 subunit knock-out (α7 KO) mice. Moreover, nicotine-treated α7 KO mice did not differ in disease response from that seen in α7 KO or WT mice exposed instead to vehicle (Hao et al., 2011). The simplest interpretation was that elimination of α7*-nAChR seemed to eliminate nicotine protection. However, the apparent absence of a difference in disease response in vehicle-treated α7 KO compared to WT mice paradoxically suggested that α7*-nAChR did not mediate natural cholinergic mechanisms involved in disease or in the response to disease (Hao et al., 2011). In addition, deeper characterization involved determinations of immune cell levels in the periphery and in the central nervous system (CNS) and of levels of expression of markers of inflammation and hyper-immunity. These levels were not statistically different for vehicle-treated α7 KO or WT animals, again suggesting lack of involvement of α7*-nAChR in natural, disease-related, cholinergic processes, which thus must involve other nAChR subtypes. Nevertheless, inflammatory and immune markers actually were suppressed in nicotine-treated α7 KO animals, although not as much as by nicotine exposure in WT mice (Hao et al., 2011). Among other things, this suggested that nAChR subtypes in addition to α7*-nAChR also must be involved in nicotine’s protective effects (Hao et al., 2011).

This intuition led to our finding that many immune system cell types express a number of nAChR subunits, most prominently α9 and β2 subunits (Hao et al., 2011), and that nicotine’s antagonism of α9*-nAChR appears to be responsible in large part for nicotine’s protective effects, which are mimicked in nAChR α9 subunit KO mice (Simard et al., 2013). This extended our knowledge about α9 subunits and nAChR containing them, previously realized to be expressed in the cochlea, olfactory epithelium and skin, but not in the CNS (Jensen et al., 2005; Lukas and Bencherif, 2006; Taly et al., 2009). Warranting emphasis, and possibly because α9 subunits are the most ancient of known, mammalian nAChR subunits, α9*-nAChR have a unique and perhaps similarly ancient pharmacological profile, in some ways akin to GABA and glycine receptors, and respond to nicotine as though it were an antagonist, rather than an agonist, whereas nicotine is an agonist mimicking effects of ACh at other nAChR subtypes. Thus, the finding is to be expected that exposure to nicotine acting as an antagonist to block α9*-nAChR function, or elimination of α9*-nAChR in a nAChR α9 subunit KO (α9 KO) mouse, leads to the same level of disease attenuation in the EAE model. In this case, elimination of α9*-nAChR eliminated nicotine protection, but this is because nicotine effectively already produces elimination of α9*-nAChR. Importantly, the findings indicate that α*-nAChR naturally contribute to disease-exacerbating effects (Simard et al., 2013).

However, we require an improved understanding of roles played by α9*-nAChR in anti- or pro-inflammatory responses, in disease-exacerbating or disease-attenuating immune or immunosuppressive responses, and in effects on those responses of nicotine exposure. Moreover, our understanding is deficient about what immune or other cell types express disease-relevant α9*-nAChR. In addition, the same questions can be raised about roles played by α7*-nAChR. For example, left open is the question about if and how α7*- and α9*-nAChR interact in modulation of inflammatory and immune processes. To address these issues, we leveraged the availability of nAChR α7/α9 subunit double knock-out (DKO) mice for study, also using direct and adoptive transfer EAE models. The results confirm disease-exacerbating roles for α9*-nAChR, apparently restricted to their expression in the periphery on immune system cells, but also reveal protective roles of α7*-nAChR expressed centrally.

## Materials and Methods

### Mice

C57BL/6J mice used as WT controls sometimes were purchased from Taconic Biosciences (Hudson, NY, USA), but they more typically were WT littermates or colony controls generated in the course of obtaining KO or DKO mice. Importantly, no differences were observed in any experiments between commercially-obtained C57BL/6 mice and the WT colony controls or littermates of the extensively backcrossed DKO mice used. The nAChR α9 subunit KO mouse model was generated by the deletion of exons 1 and 2 and their flanking intronic sequences (Genoway, Inc., Lyon, France). The embryonic stem cells used to make the transgenic mouse line were from the 129/svPas (129S2; 129/sv) strain. The α9 KO line was backcrossed at the Boys Town National Research Hospital (Omaha, NB, USA) to C57BL/6J mice (Jackson Laboratory, Bar Harbor, ME, USA) using Marker-Assisted Accelerated Backcrossing (MAX BAX; Charles River, Troy, NY, USA) until congenicity (99+%) was achieved. Details about the development of the α9 KO line have been described elsewhere (Morley et al., 2017a,b). The animals are periodically backcrossed to C57BL/6J mice to prevent genetic drift of the background strain. The absence of α9 transcript and protein in cells dissected from the retinas and inner ears of α9 KO mice has been previously reported (Smith et al., 2014; Morley et al., 2017b).

The nAChR α7 subunit KO strain was established from mice obtained from the Jackson Laboratories (JAX; RRID: IMSR_JAX:000664)^1^ as heterozygote breeders following eight generations of backcrossing at JAX. The animals were then further backcrossed for four generations at Boys Town National Research Hospital using MAX BAX, and they have since been periodically backcrossed with JAX C57BL/6J WT and nAChR α7 subunit KO animals to prevent genetic drift.

The α7/α9 DKO strain was established by breeding nAChR α9 subunit KO heterozygote females with nAChR α7 subunit KO males. Subsequent crossings used α7 or α9 female heterozygotes or α9 KO females mated with α7 or α9 male heterozygotes or KO animals.

For the studies reported here, mice were bred and housed at Boys Town National Research Hospital until maturity and shipped by overnight courier to the Barrow Neurological Institute (Phoenix, AZ, USA). The animals were bred under presumed identical conditions and tested negative for all standard viruses and parasites by IDEXX (Columbia, MO, USA).

The litter sizes for all strains varied from 5 to 9. Dams and males in our colony are typically first bred at 6–8 weeks of age, not utilized for more than two litters, and are rarely bred past the age of 12–14 weeks. Young dams and males are used for breeding to reduce the incidence of epigenetic factors that might affect the phenotype.

All animal studies were approved by Animal Care and Use Committees of the Barrow Neurological Institute or Boys Town National Research Hospital.

### Immunostaining

T (CD4^+^ or CD8^+^), monocyte/macrophage (CD11b^+^) or dendritic (CD11c^+^) cells from the spleens of WT or DKO mice were isolated using fluorescence-activated cell sorting (FACS) as described previously (Li et al., 2017). For FACS analysis, single cell suspensions (10^6^ cells) of splenocytes were stained with fluorochrome-conjugated antibodies. Targets and antibodies used (BD Biosciences, Franklin Lakes, NJ, USA) were: CD3 (145-2C11), CD4 (GK1.4), CD8 (53–6.7), CD11b (M1/70), CD11c (HL3). Flow cytometric data were collected on a FACSAria flow cytometer (BD Biosciences) and analyzed with Diva software. Isotype-matched negative control mAbs were used for all stains. Sorted cells were collected in conical tubes and then seeded on cover slips. For immunostaining, FACS-sorted cells were fixed in 4% paraformaldehyde for 10 min and washed at room temperature with phosphate-buffered saline (PBS) three times for 5 min each. A PBS-based solution containing 5% blocking serum and 0.3% Triton X-100 was applied for 1 h. Cells were incubated at 4°C overnight with primary antibodies and for 1 h at room temperature with Alexa Fluor–conjugated secondary antibodies. Cells were then washed three times for 5 min each with PBS and prepared for fluorescence microscopy as previously described (Liu et al., 2009, 2013). The following primary antibodies were used: goat anti-nAChR α9 antibody (E-17, Santa Cruz Biotechnology, Santa Cruz, CA, USA), rabbit anti-CD4 antibody (H-370, Santa Cruz Biotechnology), rat anti-CD8 antibody (2.43, Santa Cruz Biotechnology), rabbit anti-CD11b antibody (Abcam, San Francisco, CA, USA) and rabbit anti-CD11c antibody (EP1347Y, Abcam).

### EAE Induction

The mouse peptide MOG_35–55_ (myelin oliogodendrocyte glycoprotein; amino acids MEVGWYRSPFSRVVHLYRNGK) was synthesized (purity >95%) by Bio-Synthesis, Inc. (Lewisville, TX, USA). To induce EAE directly, mice were injected sub-cutaneously in the hind flank with 200 μg MOG_35–55_ peptide in complete Freund’s adjuvant (BD Biosciences) containing 500 μg of nonviable desiccated Mycobacterium tuberculosis. On the day of immunization and 2 days after, mice also were injected intravenously with 200 ng pertussis toxin (List Biological Laboratories, Campbell, CA, USA). Passive induction (adoptive transfer) EAE was initiated by transfers of splenocytes isolated from donor mice in which a direct EAE response was initiated as previously described (Huang et al., 2006; Hao et al., 2010). In brief, splenocytes were obtained from either WT or α7/α9 DKO donor mice, treated with either PBS vehicle or with nicotine starting on the day of immunization, 14 days after immunization with MOG_35–55_. Thereafter, splenocytes were cultured in the presence of 30 μg/ml MOG_35–55_ and 20 ng/ml recombinant interleukin-12 (R&D Systems). After culture for 3 days, cells were collected and washed in PBS. 1 × 10^7^ splenocytes were transferred into Rag2^−/−^ mice by intravenous injection. Recipient mice also were given 200 ng pertussis toxin intravenously on the day of cell transfer and 2 days after transfer. Whether for direct or adoptively transferred EAE, mice or recipient mice were monitored daily for symptoms scored on an arbitrary scale of 0–5 as previously described (Hao et al., 2011; Simard et al., 2013).

### Histological Analyses

At specific times after direct or adoptively transferred EAE induction, mice displaying disease scores typical of their group were anesthetized by 2% isoflurane inhalation and perfused by intracardiac puncture with 50 ml of cold PBS. Spinal cords were removed and fixed in 10% formalin/PBS. Paraffin-embedded, longitudinal sections running across the cervical enlargement were prepared and stained for infiltrating immune cells (hematoxylin and eosin), myelin (luxol fast blue) and axons (Biechowsky silver). Manual tracing was used to define the degree of inflammation, demyelination, and axonal damage across the entire spinal cord section for each mouse. Evidence of pathology was scored as follows: 0, no changes; 1, focal area involvement; 2, <5% of total myelin area involved; 3, 5%–10% of total myelin area involved; 4, 10%–20% involved area; 5, >20% of total myelin area involved (Bai et al., 2004).

### Nicotine Treatment

(−)Nicotine bitartrate was purchased from Sigma (St. Louis, MO, USA). A 100 mg/ml solution of nicotine salt in PBS, or a solution containing PBS alone, were freshly prepared and loaded into Alzet^®^ osmotic minipumps (model 1004, Durect Corporation, Cupertino, CA, USA) 24 h before pump implantation as described (Simard et al., 2013). On the day of MOG immunization, the minipumps were implanted subcutaneously on the right side of the back of the mouse and continuously delivered either PBS or nicotine salt at 12 μl/d until the end of experiments. This dosing regimen is designed to produce plasma levels of nicotine in the relevant animals of ~49 ng/ml or ~300 nM, which is comparable to concentrations of nicotine typically found in human cigarette smokers and is behaviorally relevant in mice (Matta et al., 2007).

### 7T Magnetic Resonance Imaging

Magnetic resonance imaging (MRI) was performed using a 7 Tesla, small animal, 30 cm horizontal-bore magnet and BioSpec Avance III spectrometer (Bruker Biospin MRI, Billerica, MA, USA) as previously described (Hao et al., 2010; Liu et al., 2017) Mice were under anesthesia by inhalation of 3.5% isoflurane and maintained by inhalation of 1%–2% isoflurane in 70% N_2_O and 30% O_2_ delivered via a face mask. During MRI scans, animal respiration was continually assessed by a small animal monitoring and gating system (SA Instruments, Stony Brook, NY, USA) via a pillow sensor positioned under the abdomen. Axial 2D multi-slice T2-weighted images of the brain were acquired with fat-suppressed Rapid Acquisition with Relaxation Enhancement sequence (TR = 4000 ms, effective TE = 60 ms, number of average = 4, FOV = 19.2 mm × 19.2 mm, matrix size = 192 mm × 192 mm, slice thickness = 0.5 mm, TA = 6 m 24 s). MRI data were analyzed using the MEDx3.4.3 software package (Medical Numerics, Germantown, MD, USA) on a LINUX workstation.

### *In Vivo* CNS Bioluminescence

To assess reactive oxygen species (ROS) production in brain, bioluminescence images were captured in live mice using a Xenogen IVIS200 imager (Caliper Life Sciences, Hopkinton, MA, USA) 20 min after i.p. injection of 100 μl of 50 mg/ml Luminol (Sigma-Aldrich, St. Louis, MO, USA) as we previously described (Hao et al., 2010; Simard et al., 2013). Regions of interest were selected to measure luminescence intensity in the brain. Data were collected as photons/sec/cm^2^ using Living Image^®^ software (Caliper Life Sciences, Hopkinton, MA, USA).

### Statistical Analyses

Data are presented as Mean ± SEM. Differences were considered significant at *p* < 0.05. Statistical differences among groups were evaluated by two-tailed unpaired Student’s *t*-test for two groups and by one-way analysis of variance (ANOVA) followed by Tukey *post hoc* test for three or more groups. Two-way ANOVA accompanied by a Bonferroni *post hoc* test was used for multiple comparisons. All statistical analyses were performed using Prism 5.0 software (GraphPad, San Diego, CA, USA).

## Results

### Expression Profile of nAChR α9 Subunit in Immune Cells

**Figure 1:**
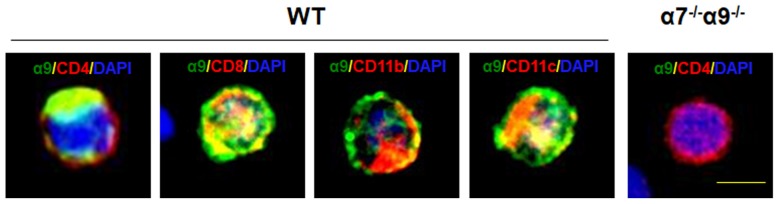
Expression of nicotinic acetylcholine receptor (nAChR) α9 subunit protein in immune cells. Immunostaining for nAChR α9 subunit protein (green) was done for the indicated, peripheral T (CD4^+^ or CD8^+^), monocyte/macrophage (CD11b^+^) or dendritic (CD11c^+^) cells labeled with cell surface marker-specific antibodies (red), and counterstained with nuclear DAPI (blue), from a wild-type (WT) mouse, or for a representative CD4^+^ T cell from a nAChR α7/α9 subunit double knock-out (DKO) animal (α7^−/−^α9^−/−^). Note that all immune cell types from the WT mouse demonstrate α9 subunit-like immunoreactivity, but that absence of immunoreactivity in the T cell from the DKO animal confirms elimination of α9 subunits, also validating specificity of the commercial antibody used. Scale bar: 5 μm.

To validate the expression of the nAChR α9 subunit gene as protein in selected immune cell types, we subjected T (CD4^+^ or CD8^+^), monocyte/macrophage (CD11b^+^) or dendritic (CD11c^+^) cells, isolated by FACS from the spleens of WT mice, to immunostaining with cell-specific markers and with an antibody against α9 subunits. All of these immune cell types display nAChR α9 subunit-like immunoreactivity (Figure 1). However, similar assessment in immune cells from nAChR α9 subunit KO mice were negative for subunit immunoreactivity (results for CD4^+^ T cells are shown; Figure 1). Interestingly, CD4^+^ T cells from nAChR α9 subunit KO mice are smaller than those from WT mice, likely indicative of immaturity.

### Nicotine Treatment Attenuates Direct EAE Severity in Both WT and α7/α9 DKO Mice, but in the Absence of Nicotine Treatment, There Is Exacerbation of Direct EAE Severity in DKO Compared to WT Animals

Our previous studies initially indicated equivalence, in disease scores and other indications of immunity and inflammation, between WT animals continuously exposed to nicotine and nAChR α9 subunit KO animals irrespective of whether they were exposed to nicotine or vehicle. Each of these three cohorts of animals had reduced severity or other indices of disease compared to vehicle-treated WT animals (Simard et al., 2013). Our initial studies using nAChR α7 subunit KO animals suggested that they did not differ from WT animals in disease score measures when treated with vehicle alone, or when α7 KO mice were exposed to nicotine, which was protective against disease in WT animals (Hao et al., 2011). Other markers of immunity and inflammation in vehicle-treated α7 subunit KO animals or WT mice were indistinguishable. However, nicotine exposure did produce attenuation of indices of immunity and inflammation in α7 KO animals, although not at the higher levels of attenuation seen in nicotine-treated WT mice. These findings suggested involvement of other nAChR subtypes in disease and protection against it. Although recognizing that there might be potential challenges in data interpretation in such studies, we nevertheless sought to determine effects on disease and nicotine protection in animals lacking both nAChR α7 and α9 subunits.

**Figure 2:**
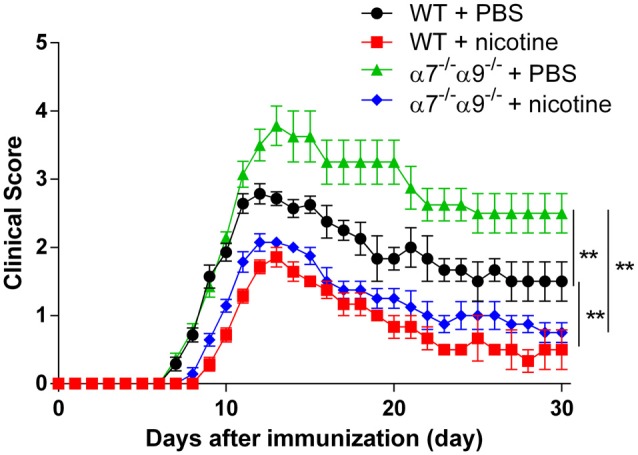
Exacerbated, direct experimental autoimmune encephalomyelitis (EAE) severity in α7^−/−^α9^−/−^ mice. EAE disease symptom evaluation was done for groups of WT or nAChR α7/α9 subunit DKO (α7^−/−^α9^−/−^) mice receiving phosphate-buffered saline (PBS) or nicotine treatment. PBS or nicotine was delivered via minipumps until the end of experiment. The minipumps were implanted subcutaneously and continuously delivered either PBS or nicotine salt at an equivalent of ~13 mg of nicotine free base/kg/day. In the absence of nicotine treatment, exacerbated EAE severity was seen in α7^−/−^α9^−/−^ mice (α7^−/−^α9^−/−^ + PBS; ▲; *n* = 8) vs. WT controls (WT + PBS; •; *n* = 7). Nicotine treatment reduced EAE severity in both α7^−/−^/α9^−/−^ (α7^−/−^/α9^−/−^ + nicotine; ⧫; *n* = 7) and WT mice (WT + nicotine; ▪; *n* = 7). Mean ± SEM; two-way analysis of variance (ANOVA); **p < 0.01.

Comparable degrees of protection against disease in the direct EAE model again are seen in WT animals and in α7/α9 DKO mice continuously exposed to nicotine (Figure 2; Table 1). These effects also are equivalent to those seen upon deletion only of α9 subunits (Simard et al., 2013). Treatment with the ligand delays the onset (by 2 days in this cohort) and attenuates the severity of disease response (by ~25%–33%) relative to the disease course in control, vehicle-treated WT animals (peak clinical scores: 2.8 ± 0.2 WT/vehicle vs. 1.9 ± 0.1 WT/nicotine, *p* < 0.01; vs. 2.1 ± 0.1 α7/α9 DKO/nicotine, *p* < 0.01; Figure 2; Table 1). However, and remarkably, direct EAE severity is elevated (by ~36%) in α7/α9 DKO mice exposed only to vehicle relative to effects in WT control animals (peak clinical scores: 2.8 ± 0.8, WT/vehicle vs. 3.8 ± 0.8 α7/α9 DKO/vehicle, *p* < 0.01; Figure 2; Table 1; Disease severity: α7/α9 DKO:saline > WT:saline > α7/α9 DKO:nicotine = WT:nicotine). Differences between groups in peak disease severity are preserved over the ~2 weeks when recovery occurs in these animals.

**Table 1:** Summary of EAE studies across nAChR subunit genotypes and treatments.

Animal (genetic status)	Treatment	Direct EAE severity (+, ++, +++ scale)	Reference(s)	Adoptive transfer EAE severity (genetic status and treatment of donor; wild-type recipient; +, ++, +++ scale)	Reference(s)
Wild-type	Saline	++	Shi et al. (2009); Hao et al. (2011); Simard et al. (2013)	++	Shi et al. (2009) This report Figure 3
	Nicotine	+	This report Figure 1	+	
α7 KO	Saline	++	Hao et al. (2011)		
	Nicotine	++			
α9 KO	Saline	+	Simard et al. (2013)		
	Nicotine	+			
α7/α9 DKO	Saline	+++	This report Figure 1	+	This report Figure 3
	Nicotine	+		+	

*Direct or adoptive EAE severity scores (scale of + to +++) are illustrated for animals with the indicated genotype and treatments (nicotine to yield blood levels of ~300 nM or saline alone) following experimental procedures as described in the “Materials and Methods” Section of this report or in the cited publication(s). Disease severity in direct EAE (++ level) is like that in wild-type (WT) animals treated with saline or in nAChR α7 subunit knock-out (α7 KO) animals irrespective of nicotine or saline treatment (WT:saline = α7 KO:saline = α7 KO:nicotine) and also is at positive control levels of disease in recipients of adoptively transferred splenocytes from WT animals treated with saline. Direct EAE disease severity is attenuated to essentially equivalent degrees in WT, α9 KO, or α7/α9 double knock-out (DKO) animals treated with nicotine, or in α9 KO animals treated with saline (WT:nicotine = α9 KO:nicotine = α7/α9 DKO:nicotine = α9 KO:saline), and there is similar attenuation of adoptively transferred EAE disease severity upon nicotine treatment of WT or α7/α9 DKO donor animals or for saline-treated α7/α9 DKO mice. Remarkably, direct EAE disease severity is worst for saline-treated α7/α9 DKO mice*.

### Brain Lesion Volume Is Larger, and Levels of Reactive Oxygen Species Are Elevated, at Peak, Directly-Induced EAE, in α7/α9 DKO Mice Compared to Results using WT Animals, but Nicotine Treatment Lessens Lesion Volume and Inflammation in Both α7/α9 DKO and WT Mice

**Figure 3:**
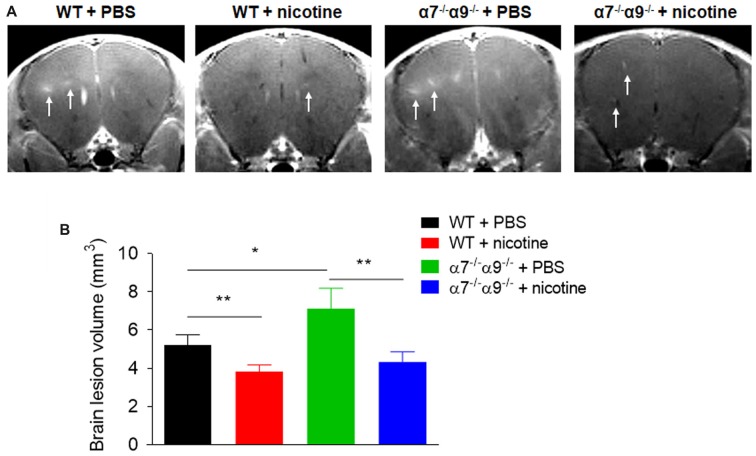
Increased brain lesion size in α7^−/−^/α9^−/−^ mice during the peak phase of direct EAE. **(A)** T1-weighted images were obtained with a 7T magnetic resonance imaging (MRI) scanner in WT or nAChR α7/α9 subunit DKO (α7^−/−^/α9^−/−^) mice receiving PBS or nicotine treatment. Mice received i.v. injections of gadolinium prior to MRI scans. Arrows indicate focal lesions in the brain and increased signal intensity on T1-weighted images. MRI scans were conducted in groups of mice (*n* = 5) at day 14 after immunization. PBS or nicotine was delivered via minipumps until the end of experiment. The minipumps were implanted subcutaneously and continuously delivered either PBS or nicotine salt at an equivalent of ~13 mg of nicotine free base/kg/day. **(B)** Bar graph shows quantified data. Mean ± SEM; one-way ANOVA; *p < 0.05, **p < 0.01.

Consistent with disease scores, MRI assessments of brain lesion volume at disease peak are reduced (~18%–27%) in nicotine-treated, WT or α7/α9 subunit DKO mice relative to effects seen in WT animals treated with vehicle (Figure 3). Lesion volumes are 5.2 ± 0.5 mm^2^ for WT/vehicle mice vs. 3.8 ± 0.3 mm^2^ for WT/nicotine animals (*p* < 0.01) and 4.3 ± 0.5 mm^2^ for α7/α9 DKO/nicotine mice (*p* < 0.01). However, compared to results in vehicle-treated WT mice, lesion volumes are elevated by about 37% in vehicle-treated α7/α9 DKO mice (7.1 ± 1.1 mm^2^), also consistent with the higher disease scores in these animals (Brain lesion volume: α7/α9 DKO:saline > WT:saline > α7/α9 DKO:nicotine = WT:nicotine).

**Figure 4:**
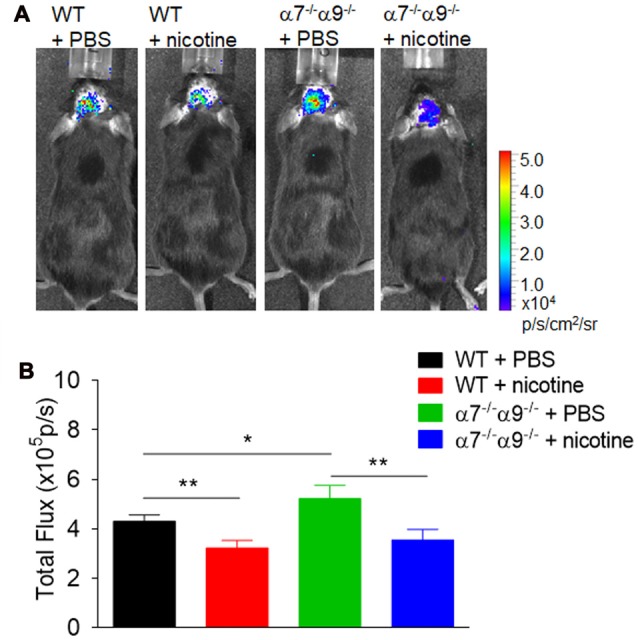
Enhanced brain inflammation on α7^−/−^α9^−/−^ mice during the peak phase of direct EAE. **(A)** Visualization and quantification of brain inflammation by *in vivo* bioluminescence imaging was conducted in WT or nAChR α7/α9 subunit DKO (α7^−/−^/α9^−/−^) mice receiving PBS or nicotine treatment. Images were captured using a Xenogen IVIS200 imager. Data were collected as photons per second per centimeter squared using Living Image software (Caliper Life Sciences). Images were obtained from groups of mice (*n* = 6) at day 14 after immunization. PBS or nicotine was delivered via mini-pumps until the end of experiment. The pumps were implanted subcutaneously and continuously delivered either PBS or nicotine salt at an equivalent of ~13 mg of nicotine free base/kg/day. **(B)** Bar graph shows quantified data. Mean ± SEM; one-way ANOVA; *p < 0.05, **p < 0.01.

Similarly, bioluminescence measurements [quantified in (photons/sec/cm^2^)/10^5^] of brain inflammation at peak disease based on ROS visualization are ~16%–26% lower, when compared to those in vehicle-treated WT animals (4.3 ± 0.3), in both nicotine-treated WT mice (3.2 ± 0.3) or nicotine-treated α7/α9 DKO animals (3.6 ± 0.4; *p* < 0.01 for both; Figure 4). Nevertheless, there is significantly elevated (~21%) CNS ROS production in vehicle-treated α7/α9 DKO (5.2 ± 0.5; *p* < 0.01 to each of the other groups (Figure 4), again reflecting higher disease scores (and brain lesion volume) in the latter group (Figure 4; Brain inflammation: α7/α9 DKO:saline > WT:saline > α7/α9 DKO:nicotine = WT:nicotine).

### Protection Against Adoptively Transferred EAE Induced by Nicotine Exposure or by Deletion of Both nAChR α7 and α9 Subunits in Donors

**Figure 5:**
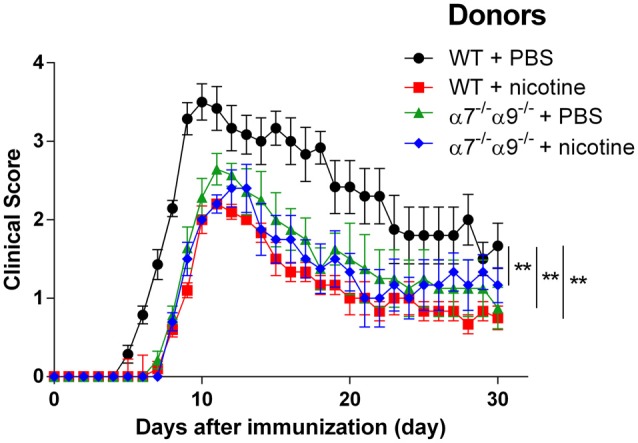
Reduced severity upon adoptive transfer EAE in Rag2^−/−^ mice receiving splenocytes from α7^−/−^α9^−/−^ mice. EAE disease symptom evaluation was done for Rag2^−/−^ mice receiving splenocytes on Day 0 from either WT or nAChR α7/α9 subunit DKO (α7^−/−^α9^−/−^) mice 14 days after the donors were immunized with MOG_35–55_. Donors also were treated starting on Day 0 with either PBS or nicotine delivered via minipumps until donation. In the absence of nicotine treatment, EAE was conferred by transfer of splenocytes from WT mice into Rag2^−/−^ recipients (WT + PBS; •), implicating immune cells originating in the periphery in the disease process (**denotes *p* < 0.01 for difference in disease score vs. the other groups). Delayed onset and lessened severity of disease signs were observed in animals receiving splenocytes from nicotine-treated DKO (α7^−/−^α9^−/−^ + nicotine; ⧫) or WT mice (WT + nicotine; ▪). Note that there is protection at the same level when splenocytes from DKO mice treated just with vehicle were transferred into Rag2^−/−^ recipients (α7^−/−^α9^−/−^ + PBS; ▲), implicating peripheral immune cells expressing α9*-nAChR and a requirement for functional α9*-nAChR expression in disease-exacerbating inflammatory and autoimmune effects. Nicotine’s effects could be due to blockade of functional α9*-nAChR on peripheral immune cells. *n* = 8. Mean ± SEM; two-way ANOVA; **p < 0.01.

α7*-nAChR are widely dispersed in central and autonomic nervous systems and are present in immune cell types. α9*-nAChR appear to be absent in the CNS but are present much more abundantly than α7*-nAChR in immune system cells (see “Discussion” Section). To determine effects of selective elimination of both subunits from immune system cells capable alone of conferring EAE to recipient mice, we also established effects of nAChR α7/α9 DKO in the adoptive transfer model of EAE. WT or DKO donor mice were treated with vehicle alone or supplemented with nicotine and inoculated with MOG peptide. Total splenocytes taken from animals at peak disease were isolated and transferred in Rag2^−/−^ recipients having the full contingent of nAChR subunits but lacking T, natural killer T, and B cells. Rag2^−/−^ recipients receiving splenocytes from vehicle-treated WT animals develop severe EAE (peak clinical score 3.5 ± 0.2; Figure 5; Table 1). However, EAE disease symptoms are attenuated (~30%) and delayed (~2–3 days) in recipients of splenocytes from nicotine-treated WT mice (2.2 ± 0.1) or from nicotine-treated DKO animals (2.4 ± 0.2; *p* < 0.01 for both; Figure 5; Table 1). A comparable degree of protection against adoptively transferred EAE is also seen for Rag2^−/−^ mice receiving splenocytes from DKO animals treated with vehicle alone (peak clinical score of 2.6 ± 0.2; *p* < 0.01 relative to WT/vehicle control; Figure 5; Table 1; Disease severity: WT:saline > α7/α9 DKO:saline = α7/α9 DKO:nicotine = WT: nicotine).

## Discussion

### Immunostaining with a Validated Antibody Confirms nAChR α9 Subunit Expression as Protein in Immune System Cells, and T Cell Maturation Appears to be Inhibited in nAChR α9 Subunit KO Mice

Results in this study indicate that many immune cell types express nAChR α9 subunits not only as mRNA, as previously shown (Hao et al., 2011), but also as protein, identified by immunostaining of peripheral (splenic) CD4^+^ and CD8^+^ T cells, CD11b^+^ monocytes/macrophages, and CD11c^+^ dendritic cells. This is consistent with other results demonstrating function of peripheral immune cell α9*-nAChR in disease model studies based on gene deletion or nicotine antagonism (Simard et al., 2013). The finding that immunostaining is absent in nAChR α7/α9 DKO mice lacking α9 subunits supports specificity of the anti-α9 subunit antibody used, which is important to demonstrate for anti-nAChR subunits (Jones and Wonnacott, 2005; Moser et al., 2007). Reciprocally, that observation also demonstrates that genetic elimination of α9 subunits translates into an absence of subunit protein. The finding that CD4^+^ T cell size is smaller in α9 KO animals is consistent with earlier studies showing that nicotine exposure, which antagonizes α9*-nAChR function, inhibits T cell differentiation and maturation, manifest as smaller size of those cells produced in thymic organ cultures and when assessed using flow cytometry (Middlebrook et al., 2000, 2002). The observation that elimination of nAChR α9 subunits (and α9*-nAChR) has anti-inflammatory and immunosuppressive effects is consistent with evident immaturity or anergy of T cells in α9 KO animals.

### α9*-nAChR Roles in Disease Exacerbating Effects

In the direct EAE model, nicotine exposure is confirmed again to delay the onset and to attenuate the severity of EAE in WT mice, and effects are shown here for the first time to be similar in nicotine-treated α7/α9 DKO animals. Brain lesion volumes and levels of ROS markers of inflammation are comparably lowered in nicotine-treated WT or α7/α9 DKO mice, paralleling as expected effects on the integrated response measured by disease score. This all is consistent with the equivalence of nicotine’s protective effects in WT or α9 KO mice, mimicking effects in vehicle-treated α9 KO mice; nicotine’s protective effects wouldn’t be expected to add to already protective effects of nAChR α9 subunit deletion in α9 KO mice (as seen in Simard et al., 2013). The interpretation of this collective body of evidence is that α9*-nAChR play roles in initiation or exacerbation of disease-relevant inflammatory and immune responses. Their antagonism by nicotine or their elimination in α9 KO mice lessens disease burden and disease-exacerbating inflammatory and immune responses.

### Multiple Targets and Disease Thresholds for Nicotine’s Protective Effects

In the directly-induced EAE model, protective effects of nicotine exposure in WT and in α7/α9 DKO mice contrast with the initially-apparent loss of nicotine’s protective effects, at least in terms of the integrated EAE disease response, in nAChR α7 KO mice (Hao et al., 2011). Why would sustained nicotine exposure not appear to be protective in α7 KO mice but protective in α7/α9 DKO mice, where its antagonism of α9*-nAChR would be expected to be redundant with α9*-nAChR elimination? One possibility is that the integrated disease response emerges once a threshold of inflammatory activity and hyper-immunity is achieved. That would imply that nicotine treatment in α7 KO mice inadequately suppresses such activity to below threshold levels. This is consistent with observations that nicotine treatment does diminish other indices of inflammation and immunity in α7 KO mice, but just not to the same degree as nicotine attenuates such responses in WT animals (Hao et al., 2011). A related interpretation is that α7*-nAChR play protective roles, and that their elimination in α7 KO animals lowers the threshold for development of disease to a level that is not overcome by nicotine exposure. Our previous studies have shown that expression levels of cytokines or other inflammatory factors change predictably in reflection of disease severity, but inflammatory status of α7/α9 DKO mice during EAE warrants further investigation, as we plan. Regardless, it is evident from nicotine’s protective roles in α7/α9 DKO mice that nicotine has additional targets other than α9*- and α7*-nAChR that are relevant to the EAE inflammatory and immune process (Simard et al., 2013).

### Newly Revealed, Natural Protective Effects of α7*-nAChR

Another important observation is that directly-induced EAE severity is elevated in vehicle-treated α7/α9 DKO mice compared to vehicle-treated WT animals. This contrasts with the aforementioned protective effects of α9 KO alone and with the initially-apparent null effect of α7 KO alone when disease manifestation is considered in comparison to that in vehicle-treated WT animals. Viewed one way, our interpretation is that there is a loss of protection against direct EAE due to elimination of naturally-functioning α7*-nAChR, even when α9*-nAChR also are deleted. Phrased differently, we postulate that upon elimination of disease-exacerbation attributable to α9*-nAChR expression, additional elimination of α7 subunits reveals protective effects of α7*-nAChR responding to natural chemical signals.

### Importance in Disease Exacerbation of Peripheral Immune Cell α9*-nAChR, and Support for Protective Roles of α7*-nAChR in the CNS

The interpretations and hypotheses advanced here thus far were tested in adoptive transfer EAE studies. Our previous work showed that nicotine treatment is comparably if not more potent in attenuating disease signs in the passive EAE model employing transfer of immune system cells from inoculated, nicotine- or vehicle-treated, WT donors to otherwise naïve, Rag2^−/−^ recipients when compared to nicotine’s protective effects against direct EAE (Shi et al., 2009). Here we show that nicotine exposure in inoculated, WT or α7/α9 DKO animals indeed protects against disease manifestation in Rag2^−/−^ recipients of immune cells from those donors compared to effects seen for transfer of immune cells from vehicle-treated WT animals. Moreover, adoptive transfer of cells from vehicle-treated α7/α9 DKO animals similarly produces attenuated disease in recipients. Our interpretation of these observations is that nicotine antagonism of α9*-nAChR, or genetic deletion of α9*-nAChR, has protective effects because they block or eliminate functional α9*-nAChR in peripheral immune cells. This finding makes sense given the known disposition of α9*-nAChR (Elgoyhen et al., 1994; Jensen et al., 2005). It is reported that nAChR α9-like immunoreactivity seems to be present in the healthy piglet or infant mouse brain (Vivekanandarajah et al., 2015, 2016). However, previous *in situ* hybridization or other measures of transcript levels (e.g., see Elgoyhen et al., 1994; Hao et al., 2011), validated by recent RefSeq data^2^, indicate that healthy mouse brain expression of nAChR α9 subunit mRNA is vanishingly low (below or at detection levels), in contrast to robust thymic (e.g., 11.423 RPKM) or immune cell levels. Furthermore, our own observations (in preparation) are entirely consistent with RefSeq^3^ data indicating that healthy human CNS levels of nAChR α9 subunits also are vanishingly low (0.154 ± 0.104 RPKM), whereas immune cell levels are substantial. Further work will ascertain whether these situations are different in diseased brain or immune system cells, across species, or across different techniques for assessment of subunit protein or message levels.

In addition, these findings indicate that elimination of nAChR α9 subunits in immune cells is protective even in the absence of immune cell α7 subunits and α7*-nAChR. Moreover, the fact that adoptively-transferred disease is attenuated in the passive EAE model rather than exacerbated, as seen in the direct EAE model, in vehicle-treated α7/α9 DKO mice, indicates that elimination of α7*-nAChR in peripheral immune cells is not damaging. This finding also supports the hypothesis/interpretation that protective roles for α7*-nAChR revealed in direct EAE studies using α7/α9 DKO mice are due to actions of those α7*-nAChR in the CNS.

### Past, Present and Future Perspectives

Delicate balancing of pro- vs. anti-inflammatory responses, and between activation or suppression of immune responses, requires cross-talk between the immune and nervous systems. Such cross-talk, and neuroimmune sharing of signaling molecules and mechanisms, even contributes to nervous system development and plasticity (Boulanger, 2009; Shatz, 2009; Besedovsky and del Rey, 2011). The chemical signaling agent, ACh, now is realized not just to have specialized activity as a neurotransmitter in the nervous system, but also to have actions in non-neuronal tissues and organs, such as in the immune system (Grando et al., 2012). There is no doubt that chemical sensing existed long before the emergence of nervous systems and concurrent specialization of chemical messengers and their targets to execute synaptic neurotransmission (Wessler and Kirkpatrick, 2008). ACh is among the primordial chemical messengers involved in chemical sensing long before they acquired more specialized functions as synaptic vesicle-packaged neurotransmitters. Even in advanced organisms with robust nervous systems, there now is solid evidence for cholinergic signaling and for the existence of enzymes that synthesize and degrade ACh in other organs and cell types, including in the immune system (Sato et al., 1999; Matsunaga et al., 2001; Kuo et al., 2002; Middlebrook et al., 2002; Kawashima et al., 2007; Fujii et al., 2008; Koval et al., 2011). Implications are that locally-synthesized and released ACh plays paracrine- or autocrine-type roles in these organs, but not discounted are possible origins of ACh from terminals of neurons providing fine innervation of those organs. However, our understanding is immature about how this “non-neuronal” cholinergic signaling is leveraged and the depth and breadth of its involvement in control of extra-neuronal cell and organ function.

α7*-nAChR were the first suggested to be involved in the so-called “cholinergic anti-inflammatory pathway” (Wang et al., 2003; Shytle et al., 2004). That implication now indeed seems validated, despite the initially-observed null effect of α7 KO on direct EAE disease signs (Nizri et al., 2009; Hao et al., 2011) which suggested that α7-nAChRs are not key players in endogenous, nAChR-dependent, immunoregulatory processes in that model. Instead, the exacerbation of direct EAE disease in α7/α9 DKO mice reveals protective roles of α7*-nAChR responding to natural cholinergic signals. This also is consistent with deeper analysis of the results using α7 KO animals showing some, but only partial, attenuation of other inflammatory and immune response indices in the presence of nicotine as opposed to vehicle, thus revealing that α7*-nAChR mediate some but not all of the effects of nicotine treatment, as one might expect for nAChR also mediating natural cholinergic signals (Hao et al., 2011). However, such roles do not seem to be due to α7*-nAChR in peripheral immune cells, based on adoptive transfer studies using α7/α9 DKO mice. Nevertheless, nicotine protection remains evident in direct EAE studies using α7/α9 DKO mice, suggesting that additional nAChR subtypes are involved, not only in its effects, but also in natural cholinergic mechanisms.

In interpretation of effects of sustained nicotine exposure, there needs to be cognizance of how those effects can deviate substantially from those of acute exposure to nicotine (Lukas, 1991, 1995, 1998; Lukas et al., 1996; Gentry and Lukas, 2002; Gentry et al., 2003). Acute exposure to nicotine or ACh stimulates nAChR channel opening (as noted above, except for α9*-nAChR, where nicotine is an antagonist rather than an agonist like ACh), but this effect is only transient. More sustained exposure can lead to functional “desensitization, ” and then to progressively “deeper” levels of “functional”/“persistent” inactivation (Lukas et al., 1996; Gentry and Lukas, 2002). These longer-term effects, just as for acute actions of ligands, differ across nAChR subtypes with regard to potency, and they likely reflect a mixture of receptor activation and inactivation (Picciotto et al., 2008). It remains controversial whether nAChR subtypes, such as α7*-nAChR, would be affected at all by nicotine at concentrations achieved via minipump delivery as used in the current studies. It also is not entirely clear whether nicotine exposure as produced in our studies would promote low level activation of specific nAChR subtypes or their functional inactivation. It also is possible that nAChR channel function is not relevant in the immune cell context, where nAChR occupancy might be adequate to alter intracellular signaling. Even for α9*-nAChR, it is not clear that nicotine exposure as in these studies would be sufficient to antagonize channel function, especially if overcome by natural cholinergic signals. Also to be defined is where and how immune cells are exposed to natural cholinergic signals. Thus, much more work is required to define mechanisms involved in the clear and indisputable protection against disease in the EAE model mediated by nicotine.

Evidence continues to build that phylogenetically-ancient α9*-nAChR naturally play disease-exacerbating roles coincident with elevated immunity and inflammation. This challenges the notion of the cholinergic anti-inflammatory system, because α9*-nAChR function seems to be anything but anti-inflammatory. Indeed, roles of α9*-nAChR in endogenous pro-inflammatory mechanisms contributing to disease initiation and evolution are likely triggered by the endogenous agonist, ACh. Yet to be tested are hypotheses that function of α9*-nAChR is required or important for maturation of T cells and perhaps other immune cell types. A consequence of nicotine antagonism-mediated α9*-nAChR functional blockade or of α9 KO producing elimination of functional α9*-nAChR, either leading to immune cell quiescence, could account for the attenuated disease response and suppression of immunity and inflammation.

As to be expected, our findings in the current study pose new questions that await future investigation. First, it remains to be determined whether deletion of specific nAChR subunits leads to compensatory changes in others, although RT-PCR studies do not indicate dramatic changes in levels of other nAChR subunit messages in α7 KO mouse immune cells, brain, or brain microglia (Hao et al., 2011). Second, there could be influences of disease state on nAChR subtype and subunit expression, also as suggested by our earlier work (Hao et al., 2011). Third, expression of nAChR that mediate natural effects of ACh and/or summed protective effects of nicotine in different immune cell types is incompletely understood. For example, it is reported that nAChR α7 and α9 subunits are expressed by CD4+ and CD8+ T cells (Qian et al., 2011), consistent with our observations (Hao et al., 2011). Moreover, regulatory T cell responses appear to be dependent on α7-nAChR (Wang et al., 2010). Infiltrating macrophages or microglia in the brain are also key players in EAE pathology, and the expression profile of α9*−nAChR and α9 subunits in these cells remains to be elucidated. Last, nAChR subtypes respond differently to nicotine and at different concentrations. Although the range of non-toxic, but behaviorally-relevant doses of nicotine that can be studied in mice or other animals *in vivo* is limited, use of other agents selective for particular nAChR subtypes could help elucidate their contributions to inflammatory and immune responses.

In summary, our observations motivate strategies involving nAChR subtype-selective compounds to modulate responses involved in inflammatory diseases. Recognizing that much more work is to be done before applying our findings to human disease conditions, we think that therapeutic possibilities are realistic and proximal. For example, α9*-nAChR of disease relevance appear to be situated in peripheral immune cells, allowing for design of ligands targeting them that would not be required to penetrate to the brain, elevating the likelihood that adverse side effects could be well-controlled. Moreover, there is limited expression of α9*-nAChR in other tissues (in olfactory epithelia and the cochlea and in some skin cells; Jensen et al., 2005; Lukas and Bencherif, 2006; Taly et al., 2009) relative to their rich expression in the immune system. Also, auditory function in α9 KO animals (but not in α9/α10 DKO mice) is like that in WT mice (Morley et al., 2017a), suggesting a low risk of non-immune system side effects if α9*-nAChR are modulated. Nicotine itself is a therapeutic candidate, as it is likely to be much safer delivered as a medicine than via use of tobacco products and has very limited abuse liability on its own in humans (West et al., 2000). There do seem to be links between elevated MS susceptibility and progression and levels of tobacco cigarette smoking (Handel et al., 2011), especially if smoking occurs in adolescence (Salzer et al., 2013). However, other studies indicate that nicotine actually could be protective in MS (Nizri et al., 2009; Shi et al., 2009); as opposed to effects seen in cigarette smokers, MS susceptibility seems to be reduced in users of snuff (Hedström et al., 2009, 2013). Nicotine exposure does not equate to cigarette smoking, which brings exposure to thousands of other harmful compounds. Thus, all things considered, α9*-nAChR are viable targets for development of superior approaches to block or control inflammation and immunity. On the other hand, α7*-nAChR activation could protect against inflammatory and autoimmune insults if activated by α7*-nAChR-selective ligands. In the long term, findings from this and other studies could alter clinical practice in the treatment of inflammatory and autoimmune diseases, such as MS.

## Author Contributions

BJM, F-DS and RJL conceived of the study and, with QL, outlined general experiments. QL executed specific methods, acquired data and engaged in initial data analysis. QL and RJL drafted the initial version of the manuscript and refined data analysis. QL, PW, BJM, F-DS and RJL contributed to data interpretation and revised the manuscript, which was progressively edited by RJL. QL, PW, BJM, F-DS, and RJL reviewed and approved the final version of the report.

## Conflict of Interest Statement

F-DS and RJL are co-holders of U.S. Patent Number 8,841,329 “Nicotinic Attenuation of CNS Inflammation and Autoimmunity.” The authors declare that the research was conducted in the absence of any commercial or financial relationships that could be construed as a potential conflict of interest. The reviewer LM and handling Editor declared their shared affiliation.

## Footnotes

^1^https://www.jax.org

^2^https://www.ncbi.nlm.nih.gov/gene/231252

^3^https://www.ncbi.nlm.nih.gov/gene/55584

## Abbreviations

α7 or α9 KO: nicotinic acetylcholine receptor α7 or α9 subunit knock-out
ACh: acetylcholine
DKO: double knock-out
EAE: experimental autoimmune encephalomyelitis
FACS: fluorescence-activated cell sorting
KO: knock-out
MOG: myelin oligodendrocyte glycoprotein
MRI: magnetic resonance imaging
MS: multiple sclerosis
nAChR: nicotinic acetylcholine receptor(s)
PBS: phosphate-buffered saline
ROS: reactive oxygen species
WT: wild-type.

## References

[B1] Bai X.-F. Li O. Zhou Q. Zhang H. Joshi P. S. Zheng X.. (2004). CD24 controls expansion and persistence of autoreactive T cells in the central nervous system during experimental autoimmune encephalomyelitis. J. Exp. Med. 200, 447–458. 10.1084/jem.20040131 15314074 PMC2211938

[B2] Besedovsky H. O. del Rey A. (2011). Central and peripheral cytokines mediate immune-brain connectivity. Neurochem. Res. 36, 1–6. 10.1007/s11064-010-0252-x 20820913

[B3] Boulanger L. M. (2009). Immune proteins in brain development and synaptic plasticity. Neuron 64, 93–109. 10.1016/j.neuron.2009.09.001 19840552

[B4] Cloëz-Tayarani I. Changeux J. P. (2007). Nicotine and serotonin in immune regulation and inflammatory processes: a perspective. J. Leukoc. Biol. 81, 599–606. 10.1189/jlb.0906544 17108054

[B5] Davidson A. Diamond B. (2001). Autoimmune diseases. N Engl J. Med. 345, 340–350. 10.1056/NEJM200108023450506 11484692

[B6] Elgoyhen A. B. Johnson D. S. Boulter J. Vetter D. E. Heinemann S. (1994). α9: an acetylcholine receptor with novel pharmacological properties expressed in rat cochlear hair cells. Cell 79, 705–715. 10.1016/0092-8674(94)90555-x 7954834

[B7] Filippini P. Cesario A. Fini M. Locatelli F. Rutella S. (2012). The yin and yang of non-neuronal α7-nicotinic receptors in inflammation and autoimmunity. Curr. Drug Targets 13, 644–655. 10.2174/138945012800399008 22300039

[B8] Franklin R. J. ffrench-Constant C. Edgar J. M. Smith K. J. (2012). Neuroprotection and repair in multiple sclerosis. Nat. Rev. Neurol. 8, 624–634. 10.1038/nrneurol.2012.200 23026979

[B9] Fujii T. Takada-Takatori Y. Kawashima K. (2008). Basic and clinical aspects of non-neuronal acetylcholine: expression of an independent, non-neuronal cholinergic system in lymphocytes and its clinical significance in immunotherapy. J. Pharmacol. Sci. 106, 186–192. 10.1254/jphs.fm0070109 18285654

[B10] Gentry C. L. Lukas R. J. (2002). Regulation of nicotinic acetylcholine receptor numbers and function by chronic nicotine exposure. Curr. Drug Targets CNS Neurol. Disord. 1, 359–385. 10.2174/1568007023339184 12769610

[B11] Gentry C. L. Wilkins L. H. Jr. Lukas R. J. (2003). Effects of prolonged nicotinic ligand exposure on function of heterologously expressed, human α4β2- and α4β4-nicotinic acetylcholine receptors. J. Pharmacol. Exper. Ther. 304, 206–216. 10.1124/jpet.102.041756 12490593

[B12] Grando S. A. Kawashima K. Kirkpatrick C. J. Meurs H. Wessler I. (2012). The non-neuronal cholinergic system: basic science, therapeutic implications and new perspectives. Life Sci. 91, 969–972. 10.1016/j.lfs.2012.10.004 23141771

[B13] Handel A. E. Williamson A. J. Disanto G. Dobson R. Giovannoni G. Ramagopalan S. V. (2011). Smoking and multiple sclerosis: an updated meta-analysis. PLoS One 6:e16149. 10.1371/journal.pone.0016149 21249154 PMC3020969

[B14] Hao J. Liu R. Piao W. Zhou Q. Vollmer T. L. Campagnolo D. I.. (2010). Central nervous system (CNS)-resident natural killer cells suppress Th17 responses and CNS autoimmune pathology. J. Exp. Med. 207, 1907–1921. 10.1084/jem.20092749 20696699 PMC2931174

[B15] Hao J. Shi F.-D. Abdelwahab M. Shi S. X. Simard A. Whiteaker P.. (2013). Nicotinic receptor β2 determines NK cell-dependent metastasis in a murine model of metastatic lung cancer. PLoS One 8:e57495. 10.1371/journal.pone.0057495 23469004 PMC3585320

[B16] Hao J. Simard A. R. Turner G. H. Wu J. Whiteaker P. Lukas R. J.. (2011). Attenuation of CNS inflammatory responses by nicotine involves α7 and non-α7 nicotinic receptors. Exp. Neurol. 227, 110–119. 10.1016/j.expneurol.2010.09.020 20932827 PMC3019302

[B18] Hedström A. K. Bäärnhielm M. Olsson T. Alfredsson L. (2009). Tobacco smoking, but not Swedish snuff use, increases the risk of multiple sclerosis. Neurology 73, 696–701. 10.1212/WNL.0b013e3181b59c40 19720976

[B17] Hedström A. Hillert J. Olsson T. Alfredsson L. (2013). Nicotine might have a protective effect in the etiology of multiple sclerosis. Mult. Scler. 19, 1009–1013. 10.1177/1352458512471879 23319071

[B19] Huang D. Shi F. D. Jung S. Pien G. C. Wang J. Salazar-Mather T. P.. (2006). The neuronal chemokine CX3CL1/fractalkine selectively recruits NK cells that modify experimental autoimmune encephalomyelitis within the central nervous system. FASEB J. 20, 896–905. 10.1096/fj.05-5465com 16675847

[B20] Jensen A. A. Frølund B. Lijefors T. Krogsgaard-Larsen P. (2005). Neuronal nicotinic acetylcholine receptors: structural revelations, target identifications, and therapeutic inspirations. J. Med. Chem. 48, 4705–4745. 10.1021/jm040219e 16033252

[B21] Jones I. W. Wonnacott S. (2005). Why doesn’t nicotinic ACh receptor immunoreactivity knock out? Trends Neurosci. 28, 343–345. 10.1016/j.tins.2005.04.010 15979499

[B22] Kawashima K. Yoshikawa K. Fujii Y. X. Moriwaki Y. Misawa H. (2007). Expression and function of genes encoding cholinergic components in murine immune cells. Life Sci. 80, 2314–2319. 10.1016/j.lfs.2007.02.036 17383684

[B23] Koval L. Lykhmus O. Zhmak M. Khruschov A. Tsetlin V. Magrini E.. (2011). Differential involvement of α4β2, α7 and α9α10 nicotinic acetylcholine receptors in B lymphocyte activation *in vitro*. Int. J. Biochem. Cell Biol. 43, 516–524. 10.1016/j.biocel.2010.12.003 21146628

[B24] Kuo Y.-P. Lucero L. Michaels J. DeLuca D. Lukas R. J. (2002). Differential expression of nicotinic acetylcholine receptor subunits in fetal and neonatal mouse thymus. J. Neuroimmunol. 130, 140–154. 10.1016/s0165-5728(02)00220-5 12225896

[B25] Lassmann H. (2013). Pathology and disease mechanisms in different stages of multiple sclerosis. J. Neurol. Sci. 333, 1–4. 10.1016/j.jns.2013.05.010 23735777

[B26] Laveti D. Kumar M. Hemalatha R. Sistla R. Naidu V. G. Talla V.. (2013). Anti-inflammatory treatments for chronic diseases: a review. Inflamm. Allergy Drug Targets 12, 349–361. 10.2174/18715281113129990053 23876224

[B27] Li M. Li Z. Yao Y. Jin W. N. Wood K. Liu Q.. (2017). Astrocyte-derived interleukin-15 exacerbates ischemic brain injury via propagation of cellular immunity. Proc. Natl. Acad. Sci. U S A 114, E396–E405. 10.1073/pnas.1612930114 27994144 PMC5255606

[B28] Liu Q. Huang Y. Xue F. Simard A. DeChon J. Li G.. (2009). A novel nicotinic acetylcholine receptor subtype in basal forebrain cholinergic neurons with high sensitivity to amyloid peptides. J. Neurosci. 29, 918–929. 10.1523/JNEUROSCI.3952-08.2009 19176801 PMC2857410

[B29] Liu Q. Jin W. N. Liu Y. Shi K. Sun H. Zhang F. (2017). Brain ischemia suppresses immunity in the periphery and brain via different neurogenic innervations. Immunity 46, 474–487. 10.1016/j.immuni.2017.02.015 28314594

[B31] Liu Q. Xie X. Lukas R. J. St John P. A. Wu J. (2013). A novel nicotinic mechanism underlies β-amyloid-induced neuronal hyperexcitation. J. Neurosci. 33, 7253–72563. 10.1523/jneurosci.3235-12.2013 23616534 PMC3865500

[B32] Lukas R. J. (1991). Effects of chronic nicotinic ligand exposure on functional activity of nicotinic acetylcholine receptors expressed by cells of the PC12 rat pheochromocytoma or the TE671/RD human clonal line. J. Neurochem. 56, 1134–1145. 10.1111/j.1471-4159.1991.tb11403.x 2002334

[B33] Lukas R. J. (1995). Diversity and patterns of regulation of nicotinic receptor subtypes. Ann. N Y Acad. Sci. 757, 153–168. 10.1111/j.1749-6632.1995.tb17471.x 7611671

[B34] Lukas R. J. (1998). “Neuronal nicotinic acetylcholine receptors,” in The Nicotinic Acetylcholine Receptors: Current Views and Future Trends, ed. Barrantes F. J. (Georgetown, TX: Springer Verlag, Berlin/Heidelberg and Landes Publishing), 145–173.

[B35] Lukas R. J. Bencherif M. (2006). “Recent developments in nicotinic acetylcholine receptor biology,” in Biological and Biophysical Aspects of Ligand-Gated Ion Channel Receptor Superfamilies, ed. Arias H. R. (Kerala, India: Research Signpost), 27–59.

[B36] Lukas R. J. Changeux J. P. Le Novere N. Albuquerque E. X. Balfour D. J. K. Berg D. K.. (1999). International Union of Pharmacology. XX. Current status of the nomenclature for nicotinic acetylcholine receptors and their subunits. Pharmacol. Rev. 51, 397–401. 10353988

[B37] Lukas R. J. Ke L. Bencherif M. Eisenhour C. M. (1996). Regulation by nicotine of its own receptors. Drug Dev. Res. 38, 136–148. 10.1002/(sici)1098-2299(199607/08)38:3/4<136::aid-ddr2>3.0.co;2-n

[B38] Matsunaga K. Klein T. W. Friedman H. Yamamoto Y. (2001). Involvement of nicotinic acetylcholine receptors in suppression of antimicrobial activity and cytokine responses of alveolar macrophages to Legionella pneumophila infection by nicotine. J. Immunol. 167, 6518–6524. 10.4049/jimmunol.167.11.6518 11714820

[B39] Matta S. G. Balfour D. J. Benowitz N. L. Boyd R. T. Buccafusco J. J. Caggiula A. R.. (2007). Guidelines on nicotine dose selection for *in vivo* research. Psychopharmacology (Berl) 190, 269–319. 10.1007/s00213-006-0441-0 16896961

[B40] Middlebrook A. J. Martina C. Chang Y. Lukas R. J. DeLuca D. (2002). Murine fetal thymus organ cultures treated with nicotine exhibit a reduction in mature thymocytes and expanded populations of immature thymocytes. J. Immunol. 169, 2915–2924. 10.4049/jimmunol.169.6.2915 12218105

[B41] Middlebrook A. J. Michaels J. Lukas R. J. DeLuca D. (2000). “Effects of nicotine exposure on developing murine and human thymocytes in fetal thumus organ culture,” in FASEB Conference on Neuroimmunology, (Copper Mountain, CO).

[B42] Morley B. J. Dolan D. F. Ohlemiller K. Simmons D. D. (2017a). Generation and characterization of α9 and α10 nicotinic acetylcholine receptor subunit knockout mice on a C57Bl/6J background. Front. Neurosci. 11:516 10.3389/fnins.2017.00516 PMC5613126 28983232

[B43] Morley B. J. Lysakowski A. Vijayakumar S. Menapace D. Jones T. A. (2017b). Nicotinic acetylcholine receptors regulate vestibular afferent gain and activation timing. J. Comp. Neurol. 525, 1216–1233. 10.1002/cne.24131 27718229 PMC6677144

[B45] Moser N. Mechawar N. Jones I. Gochberg-Sarver A. Orr-Urtreger A. Plomann M.. (2007). Evaluating the suitability of nicotinic acetylcholine receptor antibodies for standard immunodetection procedures. J. Neurochem. 102, 479–492. 10.1111/j.1471-4159.2007.04498.x 17419810

[B46] Nikoopour E. Schwartz J. A. Singh B. (2008). Therapeutic benefits of regulating inflammation in autoimmunity. Inflamm. Allergy Drug Targets 7, 203–210. 10.2174/187152808785748155 18782028

[B47] Nizri E. Irony-Tur-Sinai M. Lory O. Orr-Urtreger A. Lavi E. Brenner T. (2009). Activation of the cholinergic anti-inflammatory system by nicotine attenuates neuroinflammation via suppression of Th1 and Th17 responses. J. Immunol. 183, 6681–6688. 10.4049/jimmunol.0902212 19846875

[B48] Piao W. H. Campagnolo D. Dayao C. Lukas R. J. Wu J. Shi F. D. (2009). Nicotine and inflammatory neurological disorders. Acta Pharmacol. Sin. 30, 715–722. 10.1038/aps.2009.67 19448649 PMC4002379

[B49] Picciotto M. R. Addy N. A. Mineur Y. S. Brunzell D. H. (2008). It is not “either/or”: activation and desensitization of nicotinic acetylcholine receptors both contribute to behaviors related to nicotine addiction and mood. Prog. Neurobiol. 84, 329–342. 10.1016/j.pneurobio.2007.12.005 18242816 PMC2390914

[B50] Pierson E. Simmons S. B. Castelli L. Goverman J. M. (2012). Mechanisms regulating regional localization of inflammation during CNS autoimmunity. Immunol. Rev. 248, 205–215. 10.1111/j.1600-065X.2012.01126.x 22725963 PMC3678350

[B51] Qian J. Galitovskiy V. Chernyavsky A. I. Marchenko S. Grando S. A. (2011). Plasticity of the murine spleen T-cell cholinergic receptors and their role in *in vitro* differentiation of naive CD4 T cells toward the Th1, Th2 and Th17 lineages. Genes Immun. 12, 222–230. 10.1038/gene.2010.72 21270829

[B52] Salzer J. Hallmans G. Nyström M. Stenlund H. Wadell G. Sundström P. (2013). Smoking as a risk factor for multiple sclerosis. Mult. Scler. 19, 1022–1027. 10.1177/1352458512470862 23257617

[B53] Sato K. Z. Fujii T. Watanabe Y. Yamada S. Ando T. Kazuko F.. (1999). Diversity of mRNA expression for muscarinic acetylcholine receptor subtypes and neuronal nicotinic acetylcholine receptor subunits in human mononuclear leukocytes and leukemic cell lines. Neurosci. Lett. 266, 17–20. 10.1016/s0304-3940(99)00259-1 10336173

[B54] Shatz C. J. (2009). MHC class I: an unexpected role in neuronal plasticity. Neuron 64, 40–45. 10.1016/j.neuron.2009.09.044 19840547 PMC2773547

[B55] Shi F. D. Piao W. H. Kuo Y. P. Campagnolo D. I. Vollmer T. L. Lukas R. J. (2009). Nicotinic attenuation of central nervous system inflammation and autoimmunity. J. Immunol. 182, 1730–1739. 10.4049/jimmunol.182.3.1730 19155522

[B56] Shytle R. D. Mori T. Townsend K. Vendrame M. Sun N. Zeng J.. (2004). Cholinergic modulation of microglial activation by α7 nicotinic receptors. J. Neurochem. 89, 337–343. 10.1046/j.1471-4159.2004.02347.x 15056277

[B57] Simard A. R. Gan Y. St-Pierre S. Kousari A. Patel V. Whiteaker P.. (2013). Differential modulation of experimental autoimmune encephalomyelitis by α9*- and β2*-nicotinic acetylcholine receptors. Immunol. Cell Biol. 91, 195–200. 10.1038/icb.2013.1 23399696 PMC3596513

[B58] Smith M. L. Souza F. G. O. Bruce K. S. Strang C. E. Morley B. J. Keyser K. T. (2014). Acetylcholine receptors in the retinas of the α7 nicotinic acetylcholine receptor knockout mouse. Mol. Vis. 20, 1328–1356. 25352741 PMC4169779

[B59] Taly A. Corringer P.-J. Guedin D. Lestage P. Changeux J.-P. (2009). Nicotinic receptors: allosteric transitions and therapeutic targets in the nervous system. Nat. Rev. Drug Discov. 8, 733–750. 10.1038/nrd2927 19721446

[B60] Tracey K. J. (2009). Reflex control of immunity. Nat. Rev. Immunol. 9, 418–428. 10.1038/nri2566 19461672 PMC4535331

[B61] Ulloa L. (2005). The vagus nerve and the nicotinic anti-inflammatory pathway. Nat. Rev. Drug Discov. 4, 673–684. 10.1038/nrd1797 16056392

[B62] Viganò S. Perreau M. Pantaleo G. Harari A. (2012). Positive and negative regulation of cellular immune responses in physiologic conditions and diseases. Clin. Dev. Immunol. 2012:485781. 10.1155/2012/485781 22548114 PMC3324270

[B63] Vivekanandarajah A. Chan Y. L. Chen H. Machaalani R. (2016). Prenatal cigarette smoke exposure effects on apoptotic and nicotinic acetylcholine receptor expression in the infant mouse brainstem. Neurotoxicology 53, 53–63. 10.1016/j.neuro.2015.12.017 26746805

[B64] Vivekanandarajah A. Waters K. A. Machaalani R. (2015). Postnatal nicotine effects on the expression of nicotinic acetylcholine receptors in the developing piglet hippocampus and brainstem. Int. J. Dev. Neurosci. 47, 183–191. 10.1016/j.ijdevneu.2015.09.007 26440997

[B66] Wang H. Yu M. Ochani M. Amella C. A. Tanovic M. Susarla S.. (2003). Nicotinic acetylcholine receptor α7 subunit is an essential regulator of inflammation. Nature 421, 384–388. 10.1038/nature01339 12508119

[B65] Wang D. W. Zhou R. B. Yao Y. M. Zhu X. M. Yin Y. M. Zhao G. J.. (2010). Stimulation of α7 nicotinic acetylcholine receptor by nicotine increases suppressive capacity of naturally occurring CD4^+^CD25^+^ regulatory T cells in mice *in vitro*. J. Pharmacol. Exp. Ther. 335, 553–561. 10.1124/jpet.110.169961 20843956

[B67] Weiner H. L. Selkoe D. J. (2002). Inflammation and therapeutic vaccination in CNS diseases. Nature 420, 879–884. 10.1038/nature01325 12490962

[B68] Wekerle H. (1998). The viral triggering of autoimmune disease. Nat. Med. 4, 770–771. 10.1038/nm0798-770 9662363

[B69] Wessler I. Kirkpatrick C. J. (2008). Acetylcholine beyond neurons: the non-neuronal cholinergic system in humans. Br. J. Pharmacol. 154, 1558–1571. 10.1038/bjp.2008.185 18500366 PMC2518461

[B70] West R. Hajek P. Foulds J. Nilsson F. May S. Meadows A. (2000). A comparison of the abuse liability and dependence potential of nicotine patch, gum, spray and inhaler. Psychopharmacology (Berl) 149, 198–202. 10.1007/s002130000382 10823399

